# Stimuli-Responsive Gold Nanocages for Cancer Diagnosis and Treatment

**DOI:** 10.3390/pharmaceutics14071321

**Published:** 2022-06-22

**Authors:** Chunming Li, Tengyue Zhao, Lixian Li, Xiaogang Hu, Chao Li, Wanyi Chen, Yurong Hu

**Affiliations:** 1 Department of Pharmacy, Chongqing University Cancer Hospital, No. 181 Hanyu Road, Chongqing 400030, China; lchm@cqu.edu.cn (C.L.); lilixian@cqu.edu.cn (L.L.); hxgcq1987@126.com (X.H.); lichaolucy@sina.com (C.L.); 2 School of Pharmaceutical Sciences, Zhengzhou University, No. 100 Kexue Avenue, Zhengzhou 450001, China; waty1573121234@163.com

**Keywords:** gold nanocages, stimuli-responsive, controlled release, cancer, diagnosis, treatment

## Abstract

With advances in nanotechnology, various new drug delivery systems (DDSs) have emerged and played a key role in the diagnosis and treatment of cancers. Over the last two decades, gold nanocages (AuNCs) have been attracting considerable attention because of their outstanding properties. This review summarizes current advancements in endogenous, exogenous, and dual/multi-stimuli responsive AuNCs in drug delivery. This review focuses on the properties, clinical translation potential, and limitations of stimuli-responsive AuNCs for cancer diagnosis and treatment.

## 1. Introduction

**Scheme 1:**
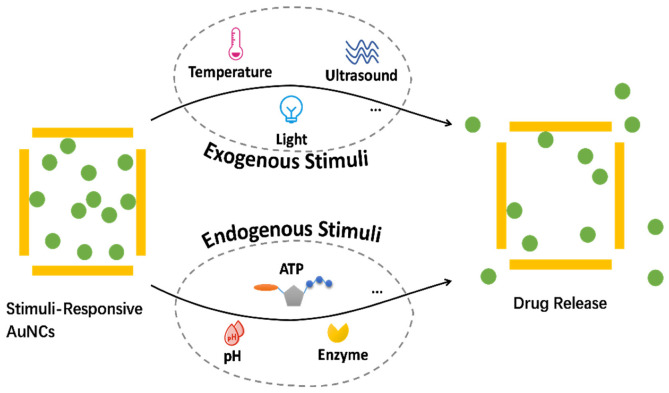
Schematic illustration of stimuli-responsive AuNCs.

Over the past decades, nanotechnology has been increasingly used in the diagnosis and treatment of cancer. To date, several types of nanocarriers have been engineered for drug delivery in oncology, including dendrimers, polymeric micelles, liposomes, metal nanoparticles, and cell membrane-coated nanoparticles [1,2,3]. The unique physicochemical characteristics of AuNCs, including adjustable particle size and pore volume, large specific surface area, outstanding biocompatibility, and excellent drug-loading capacity, make them a popular candidate for drug delivery [4,5,6]. As a result, various studies have exploited AuNCs to create new stimuli-responsive nanocarriers (Scheme 1). To entrap cargo by blocking the pores of AuNCs, a variety of biocompatible gatekeepers have already been used. The existence of at least one stimulus causes cargo to be released. The drug-delivery aspect of AuNCs has already been covered in a few reviews [7,8,9,10,11,12]. However, to the best of our knowledge, none of them are specifically intended for stimuli-responsive AuNCs. This review explores the chemistry underpinning the fabrication of stimuli-responsive AuNCs and their role in controlled drug release in response to certain stimuli. The review also summarizes obstacles in the way of AuNC-based stimuli-responsive DDSs and broadens the scope by discussing the current advancements in the field and various stimuli-responsive mechanisms.

## 2. Endogenous Stimuli-Responsive AuNCs

Various endogenous stimuli, such as low interstitial pH, high concentrations of glutathione, or high levels of specific enzymes, have been utilized to control the biodistribution and drug release of nanocarriers [13,14,15,16,17,18,19]. This section focuses on AuNC-based DDSs triggered by endogenous stimuli. Because they do not need external triggers to activate the drug release, endogenous stimuli-responsive AuNCs avoid any invasive procedures. In the ideal case of cancer treatment, internal stimuli for drug release are superior to external stimuli. Firstly, stimuli at specific pathological target sites tend to result in precise drug release at the correct location, whereas external stimuli may affect larger physiological volumes and thus lack site specificity. Secondly, internal stimuli-responsive DDSs do not require specialized types of equipment to generate stimulation, thus reducing treatment costs.

### 2.1. Enzyme-Responsive AuNCs

**Figure 1:**
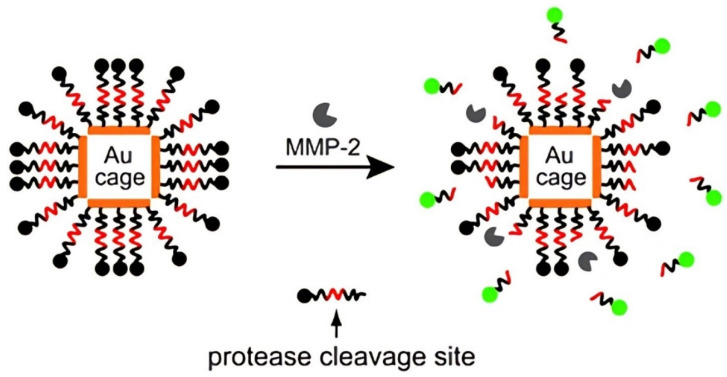
Schematic illustration of enzyme-responsive AuNCs conjugated with FITC-labeled peptides. Reprinted/adapted with permission from Ref. [30]. Copyright 2011, The Royal Society of Chemistry.

Enzymes function as biocatalysts in metabolic processes [20]. Cancers are associated with abnormal or dysregulated expressions of enzymes, e.g., esterases, glycosidases, or proteases are often expressed at higher levels in tumor cells than in normal cells [21]. Abnormal enzyme content or activity can be used in the diagnosis of some diseases [22,23]. In addition, stimuli-responsive drug carriers can be prepared by utilizing high concentrations of enzymes in many target tissues [24,25]. Enzyme-responsive nanocarriers possess specific molecules that can be identified and degraded by overexpression of enzymes in the extracellular or intracellular environments of tumors [26]; Cathepsin B [27], hyaluronidase (HAase) [28], and matrix metalloproteinases (MMPs) [29] are enzymes frequently used as triggers. MMPs are enzymes related to invasion and metastasis of cancer cells. As shown in Figure 1, Xia et al. [30] modified the surface of AuNCs with an FITC-labeled peptide (fluorescein isothiocyanate-GKG*PLGVR*GC-NH_2_) that can be cleaved by MMP. In the absence of MMP-2 protease, the fluorescence of the FITC was quenched. However, in the presence of MMP-2, the peptide was cleaved and the dye molecule was released from the surface of the AuNCs, re-emitting fluorescence. If the localized surface plasmon resonance (LSPR) peak of the AuNCs is far away from the emission peak of the dye, the fluorescence emitted by the released dye can be detected with high sensitivity. Fluorescence microscopy and spectroscopy studies revealed that the nanoprobe could respond to low enzyme concentrations and low enzymatic activity. Because the LSPR peaks of the AuNCs can be continuously tuned in the near-infrared (NIR) range, different combinations of dyes and AuNCs can be flexibly chosen to construct probes most suitable for in vivo imaging of cellular and enzymatic activities. This approach can potentially be used for the controlled release of DDSs in response to MMP concentrations.

### 2.2. pH-Responsive AuNCs

It is well-known that the pH of most tumor tissues (~6.8) is more acidic than that of normal tissues (~7.4). Additionally, endocytic vesicles have a pH of 5.5–6.0 in endosomes and 4.5–5.0 in lysosomes [31,32]. Therefore, pH-responsive nanocarriers can be designed by using pH differences to control drug release [33].

Hu et al. [34] fabricated a new type of double-walled Au/SiO_2_ nanoparticles by coating the surface of AuNCs with SiO_2_. The porous SiO_2_ shell ensured the stability of the AuNCs and enhanced the drug-loading capacity. The Tat peptide conjugated to the surface of Au/SiO_2_ nanocomposites had positive potential and the internalization-assistance property, which significantly improved the uptake efficiency of nanocomposites by MCF-7 cells, allowing for the site-specific localization of therapeutic drugs. The Au/SiO_2_ nanoparticles slowly released doxorubicin (DOX) under normal physiological conditions (PBS, pH = 7.4), but DOX was released more rapidly in the simulated intracellular environment of cancer cells (PBS, pH = 5.0) and the release trend was stable throughout the incubation time. Consequently, after incubation for 22 h, a large amount of DOX (46.2%) was released. Under acidic conditions, the relatively positive potential of DOX molecules weakened the electrostatic adsorption force, which may be the reason for the increased drug-release rate. Based on its ingenious design, this study increases our understanding of how to build intelligent nanoparticles for better cancer treatments.

### 2.3. ATP-Responsive AuNCs

Adenosine triphosphate (ATP), one of the most important biological molecules, provides energy for most biological processes. Accumulating evidence has shown that ATP is associated with various pathological processes, including uncontrolled tumor growth and chemotherapy resistance, thus becoming an important marker to distinguish cancer cells from normal cells [35,36,37]. Mainly due to the excessive glycolysis, tumor cells have high concentrations of ATP (1–10 × 10^−3^ M) [38,39].

**Figure 2:**
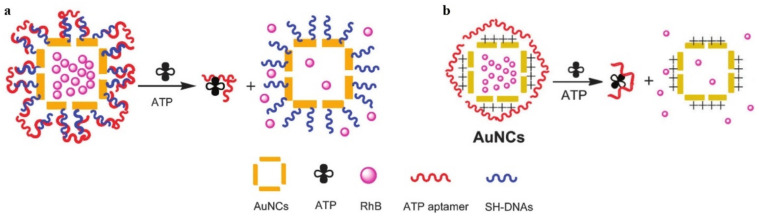
Schematic illustration of two kinds of ATP-responsive AuNCs for ATP detection. In the presence of ATP, specific recognition reactions selectively triggered the release of the encapsulated RhB. (**a**) ATP aptamers were capped on the surface of AuNCs via oligonucleotides; (**b**) ATP aptamers were capped on the surface of AuNCs via electrostatic interactions. Reprinted/adapted with permission from Refs. [40,41]. Copyright 2012 and 2015, respectively, The Royal Society of Chemistry.

Wang et al. [40] fabricated a kind of ATP-responsive controlled-release drug delivery system (DDS) based on AuNCs, which were functionalized with two types of thiol-modified single-stranded oligonucleotides (SH-DNAs) via Au-thiolate bonds on the surface. The bases of the two immobilized SH-DNA are somewhat complementary to the two ends of the ATP aptamer. Therefore, the addition of ATP caused the removal of ATP aptamers from the surface of AuNCs, allowing the cargo molecule, Rhodamine B (RhB), to escape through the pores of AuNCs (Figure 2a). The as-prepared nanodevice can be significantly disassembled by ATP activation, enabling the controlled release of ATP-activated drugs in cancer cells while reducing toxicity to normal cells. They further fabricated magnetic nanoparticles combined with RhB-loaded aptamers-AuNCs (denoted Apt-AuNC-MNPs sensing device). To examine the specificity of this sensing device, tests were conducted with ATP and its analogs such as uridine triphosphate (UTP), guanosine triphosphate (GTP), and cytosine triphosphate (CTP). Under the same test parameters, aqueous samples containing 1.0 × 10^−5^ M ATP, UTP, GTP, and CTP were analyzed with the nanodevice, among which, the presence of ATP resulted in the maximum release of fluorescent molecules, demonstrating the excellent specificity of the sensing device.

In another study, Wang et al. [41] further developed a novel controlled-release system based on positively charged AuNCs capped with molecular gates of bioresponsive aptamers. The surfaces of AuNCs were modified with cationic poly(diallyl dimethylammonium) chloride to obtain positively charged AuNCs. Under electrostatic interaction, aptamers formed molecular gates on the surface of AuNCs. Due to the specific recognition response between ATP and ATP aptamers, after the target ATP is mixed with the capped AuNCs, the ATP aptamers can be removed from the surface of the AuNCs, resulting in the opening of the pores and the release of the cargo molecules (Figure 2b). In the presence of specific targets, the aptamers assembled on the surface of AuNCs via electrostatic interaction were removed more easily than those assembled via DNA hybridization. Thus, this system was capable of detecting trace amounts of target molecules with high sensitivity and selectivity.

### 2.4. MicroRNA-Responsive AuNCs

MicroRNAs (miRNAs) are small non-coding single-stranded RNA molecules involved in the regulation of gene expression [42,43]. MiRNAs are often misregulated in tumor tissues [44,45], so they can be used as biomarkers for the early diagnosis of tumors [46,47]. However, there are few studies on the use of miRNAs for tumor diagnosis and therapy.

**Figure 3:**
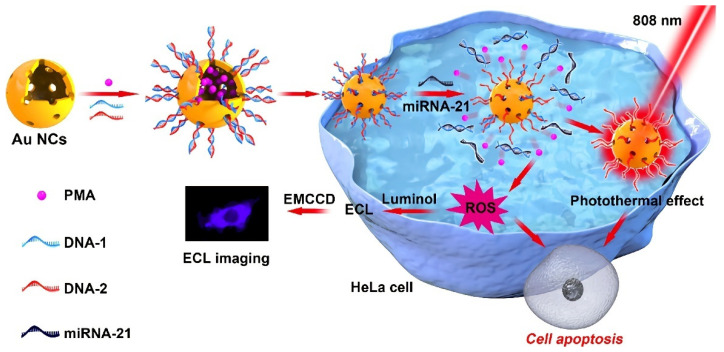
Schematic diagram of the microRNA-responsive AuNCs@PMA probe for controlled drug release, ECL imaging, and combined therapy. Reprinted/adapted with permission from Ref. [48]. Copyright 2019, American Chemical Society.

Zhang et al. [48] developed an electrochemiluminescence (ECL) microscope with diagnostic and therapeutic functions. In this study, DNA gate-2 was first modified on the surface of AuNCs through gold–sulfur bonds, and then DNA gate-1 was partially hybridized with DNA gate-2 to form a DNA gate, and phorbol 12-myristate 13-acetate (PMA) was encapsulated in the cavities of AuNCs to obtain the AuNCs@PMA probe. After the probe was endocytosed by HeLa cells, the miRNA-21 in HeLa cells was completely hybridized with DNA-1, making it detach from AuNCs, releasing PMA from the AuNCs@PMA probe, and inducing HeLa cells to produce reactive oxygen species (ROS). In addition, ROS and the photothermal effect of AuNCs had a significant lethal effect on HeLa cells. Moreover, the H_2_O_2_ component of ROS could react with luminol solution for ECL imaging (Figure 3). However, in normal cells, the DNA gate could not be opened due to the absence of miRNA-21, so the ECL signal was negative. The AuNCs@PMA probe is anticipated to play an important role in future cancer diagnosis and treatment due to its excellent performance.

## 3. Exogenous Stimuli-Responsive AuNCs

Compared with AuNCs that respond to endogenous stimuli, exogenous stimulus-responsive AuNCs have the potential to reduce interindividual variability because drug release is regulated by external variables and can be precisely controlled in these systems. These external stimuli include temperature, light, and ultrasound.

### 3.1. Temperature-Responsive AuNCs

**Figure 4:**
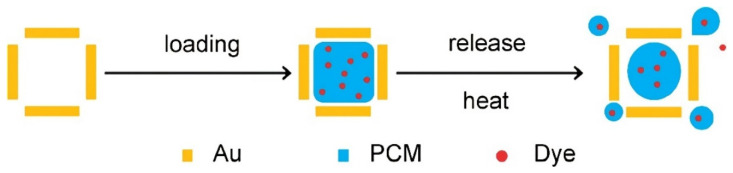
Schematic illustration of temperature-responsive AuNCs loaded with dye-doped PCM. Reprinted/adapted with permission from Ref. [52]. Copyright 2011, American Chemical Society.

Heating is the most straightforward method to achieve controlled release, and the simplest method to evaluate the effect of controlled release. On the other hand, temperature increases caused by pathological stimuli at the lesion site can also be used to trigger drug release. Typically, solid tumors have a microenvironment that is 1–2 °C warmer than that of healthy tissues [49,50]. Poly-N-isopropylacrylamide (PNIPAAm) and its derivatives are among the most extensively used thermosensitive materials as they may change their structure from contraction to expansion (and vice versa) in response to temperature changes [51]. On the other hand, phase-change materials (PCMs) can be used as a new class of thermosensitive materials for controlled drug release. At the phase-transition temperatures, PCMs undergo reversible phase transitions. Therefore, PCMs can be used as a multifunctional platform to encapsulate various therapeutic agents, which can be released only when the PCMs melt. The encapsulation of PCM into AuNCs for controlled drug release was first reported in 2011 [52]. In this study, Rhodamine 6G and methylene blue were used as simulants of chemotherapeutic drugs, which were fully mixed with molten 1-tetradecyl alcohol (1-TD, a kind of PCM with a melting point of 38–39 °C) and diffused into the cavities of AuNCs. At temperatures below its melting point, 1-TD could effectively encapsulate drug molecules within AuNCs; when the temperature was raised above the melting point of 1-TD, these drug molecules could be released from AuNCs with the melted 1-TD (Figure 4). In short, the drug-release process from nanosystems can be controlled by this simple and universal method.

However, it is difficult to accurately manage drug release by relying solely on changes in internal temperature. Therefore, in most cases, a rapid increase in lesion temperature caused by other indirect external stimuli (such as ultrasound and near-infrared light, which will be discussed in depth in the following sections) is more effective in triggering drug release.

### 3.2. Light-Responsive AuNCs

Among various external stimuli, light is often employed as a trigger for DDSs due to its non-invasiveness, long-range responsiveness, and strong controllability [53,54]. In recent years, a wide range of light-responsive nanoparticles have been developed to achieve on-demand drug release in response to irradiation with different wavelength ranges (ultraviolet [55], visible [56], and near-infrared light [57]). However, ultraviolet and visible light are considered unsuitable for therapy-related in vivo applications due to their poor penetration, while NIR light is promising in achieving on-demand drug release because of its safety and deeper tissue penetration [58,59,60,61,62].

**Figure 5:**
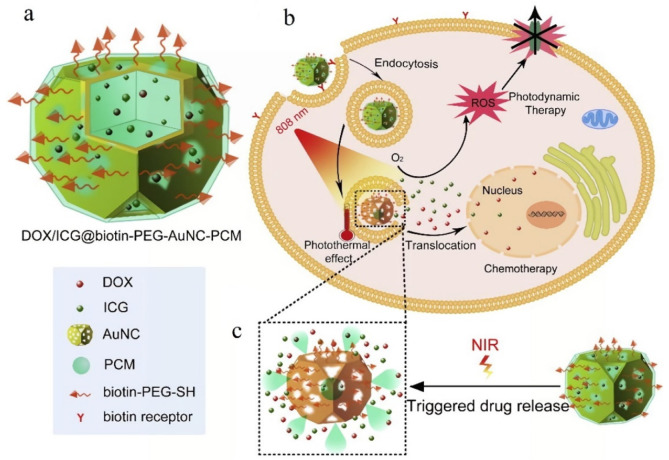
Schematic illustration of (**a**) the structure of DOX/ICG@biotin-PEG-AuNC-PCM nanosystem; (**b**) cellular uptake of DOX/ICG@biotin-PEG-AuNC-PCM by MCF-7/ADR through receptor-mediated endocytosis, intracellular release of ICG and DOX for chemo- and photodynamic therapy; (**c**) NIR-triggered drug release from DOX/ICG@biotin-PEG-AuNC-PCM. Reprinted/adapted with permission from Ref. [66]. Copyright 2017, Acta Materialia Inc. Published by Elsevier Ltd.

AuNCs possess plasmonic properties and are very efficient at converting NIR light to thermal energy, which can trigger AuNCs to release drugs (such as DOX [51], H_2_SeO_3_ [63], Ca^2+^ [64], and radical source [65]) without adding NIR dyes, thus reducing the complexity of the drug-release systems. Yu et al. [66] designed a NIR-triggered co-release system of DOX and indocyanine green (ICG) by combining the photothermal capability of AuNCs with the temperature-sensitive phase-transition feature of 1-TD. The DOX/ICG@biotin-PEG-AuNC-PCM nanosystem was generated by filling the cavities of AuNCs with ICG, DOX, and 1-TD, and then modifying the surface with biotinylated-polyethylene glycol (biotin-PEG) via gold–sulfur bonds. At 40 °C or under 808 nm NIR irradiation of 2.5 W/cm^2^, the co-release of DOX and ICG from nanosystems in PBS was significantly faster than at 37 °C (e.g., 67.27% or 80.31% vs. 5.57% of DOX, 76.08% vs. 3.83% of ICG for 20 min). Under the irradiation of NIR light, the AuNCs generated heat, which triggered the simultaneous release of ICG and DOX, and enhanced the distribution of DOX in the nuclei. The released ICG acted as a photosensitizer to generate reactive oxygen species for photodynamic therapy, showing the potential to enhance MDR cancer therapy through the synergistic effect of photothermal therapy, chemotherapy, and photodynamic therapy (Figure 5).

By loading aluminum phthalocyanine (AlPcS) into AuNCs, Xu et al. [67] prepared AlPcS–AuNC conjugates. After the conjugates entered target cells, they were subjected to a femtosecond pulsed laser at 780 nm (100 fs, 80 MHz, 50 W/cm^2^, 20 s), which triggered the explosion of the gold nanocages and decomposed them into fragments of different sizes, thereby fully releasing aluminum phthalocyanine and exerting its effect of photodynamic therapy (PDT). However, a 780 nm continuous-wave laser with the same power and duration did not cause the gold nanocages to rupture. In addition, based on the difference in transient life between free and conjugated forms of aluminum phthalocyanine, the drug-release kinetics were studied by the real-time imaging function of time-resolved transient absorption spectra. This study would also provide new references for related research in nanodrug delivery.

**Figure 6:**
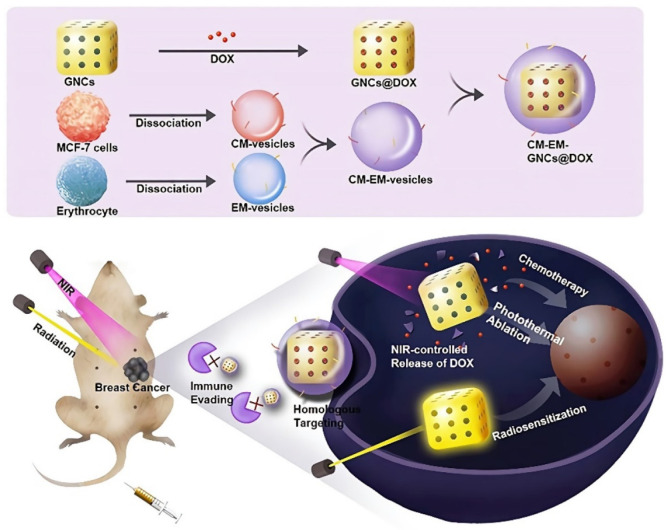
Schematic illustration of CM-EM-GNCs@DOX with NIR-responsive drug release properties for combined photothermal/radiation/chemotherapy of breast cancer. Reprinted/adapted with permission from Ref. [68]. Copyright 2020, Sun et al.

In 2020, Sun et al. [68] developed DOX-loaded erythrocyte-cancer cell hybrid membrane-coated gold nanocages (CM-EM-GNCs@DOX) for combined photothermal/radiation/chemotherapy of breast cancer. The CM-EM-GNCs@DOX demonstrated excellent photothermal transition efficiency and NIR-responsive drug-release properties (Figure 6). In the first 9 h, up to about 80% of DOX was released from GNCs@DOX nanoparticles without CM-EM coating. Within 24 h after CM-EM coating, the leakage of DOX was only less than 20%, demonstrating that the cell membrane coating could successfully improve the stability of DDSs in the physiological environment. Under the irradiation of NIR light at 808 nm and 50 mW/cm^2^, the outer cell membrane was disrupted by the photothermal effect and the release of DOX from CM-EM-GNCs@DOX was significantly increased with a release rate exceeding 80%. NIR light-induced drug release from CM-EM-GNCs@DOX nanoparticles facilitates precision chemotherapy. Additionally, during in vitro and in vivo investigations, CM-EM-GNCs@DOX demonstrated integrated photothermal/radio/chemotherapeutic efficacy with low adverse effects. This nanoplatform introduces a novel concept and strategy for cancer treatment.

In another study, He et al. [69] developed a novel nanosystem for NIR-triggered drug release and combined chemo–photothermal therapy employing DOX-loaded and thermosensitive liposome-coated AuNCs (Lipos-AuNC-DOX, LAD). Liposome coating increased cellular uptake of LAD while preventing drug leakage in the blood circulation. More notably, under NIR irradiation, LAD displayed controllable photothermal conversion and generated mild heat. As a result, regulating thermogenesis could not only efficiently initiate the phase transition of the lipid layer, resulting in the release of DOX, but also promote the heat-stress injury of cancer cells.

### 3.3. Ultrasound-Responsive AuNCs

Ultrasound, which has radiation force, and mechanical or thermal effects, can be regulated in a remote, non-invasive and spatiotemporal way to control the release of drugs [70,71,72,73].

In particular, high-intensity focused ultrasound (HIFU) has been found to be a promising trigger for controlling drug release due to its ultra-high energy and millimeter-scale focusing ability [74,75,76]. In addition, HIFU can penetrate deeper into soft tissues than NIR light, so it may be a more effective external trigger for rapid and on-demand drug release [77]. Moon et al. [52] fabricated an HIFU-responsive drug delivery system by encapsulating PCMs and biological or chemical effectors into the interior of AuNCs. When exposed to HIFU, the PCM would melt and flow out through the pores of the AuNCs, releasing the entrapped molecules. In addition, the release behavior could be adjusted by altering the power or duration of HIFU. The new hybrid system, composed of AuNCs and PCM, can also be endowed with the functions of molecular imaging, chemo-, and photothermal therapy for the diagnosis and treatment of tumors.

**Figure 7:**
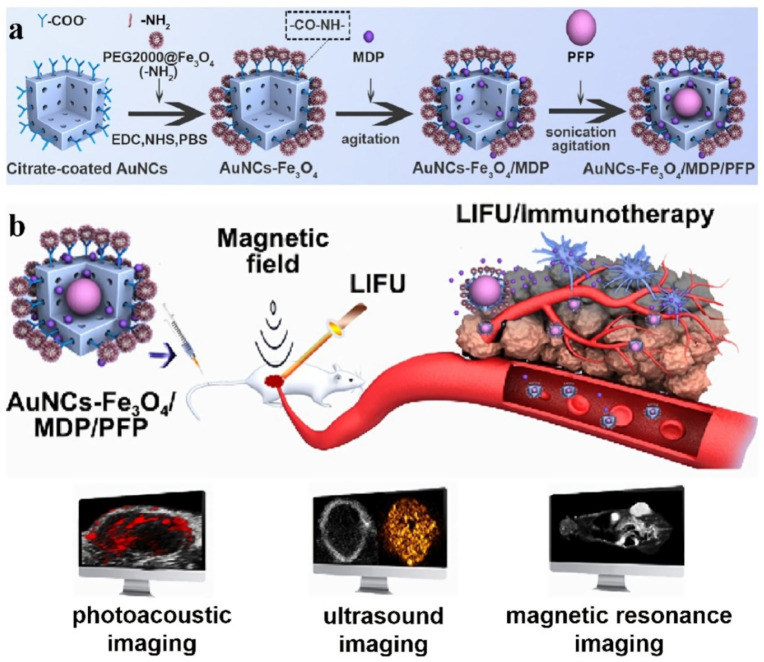
(**a**) Fabrication process of AuNCs-Fe_3_O_4_/MDP/PFP; (**b**) schematic illustration of AuNC-Fe_3_O_4_/MDP/PFP nanoparticles for multimodal imaging-guided LIFU/immunosynergistic therapy of retinoblastoma. Reprinted/adapted with permission from Ref. [80]. Copyright 2020, American Chemical Society.

Compared with HIFU, low-intensity focused ultrasound (LIFU) is considered as an effective tool for reducing damage to surrounding normal tissues. Unlike HIFU, the dominant function of LIFU is its mechanical effect, in which bubbles in the sound field are induced to vibrate, expand, and collapse. Based on these advantages, LIFU can be used as a switch to modulate drug release [78,79]. Wang et al. [80] coupled Fe_3_O_4_ on the surfaces of AuNCs to obtain AuNCs-Fe_3_O_4_ nanoparticles, in which muramyl dipeptide (MDP) and perfluoropentane (PFP) were encapsulated to generate LIFU-responsive AuNCs-Fe_3_O_4_/MDP/PFP nanocomposites for a combination of LIFU/immunotherapy and multimodal imaging (photoacoustic imaging, ultrasound imaging, and magnetic resonance imaging) of cancers. PFP underwent a liquid-to-gas phase change upon irradiation with LIFU to release MDPs, which stimulated dendritic cells to detect and kill tumor cells. The drug-release rate of nanocomposites was very slow in the absence of LIFU, but increased rapidly under LIFU irradiation (Figure 7). Furthermore, the larger bubbles induced by the phase transition formed gaps between the AuNCs-Fe_3_O_4_ shells, enabling more efficient drug release from the nanocomposites. The LIFU/immunosynergistic therapy was successful in reducing tumor growth and preventing recurrence. Furthermore, the nanoplatform has been proven to have great biosafety and biocompatibility in vitro and in vivo, so it has great potential for translation to the clinic.

## 4. Dual/Multi-Stimuli-Responsive AuNCs

In addition to single-stimulus-responsive AuNCs, other AuNC-based nanocarriers have also been investigated, which can respond to dual or multiple stimuli in order to better respond to the environment of cancer cells and achieve higher specificity and efficacy. These stimuli can be endogenous, exogenous, or an integration of both.

### 4.1. pH- and Light-Responsive AuNCs

**Figure 8:**
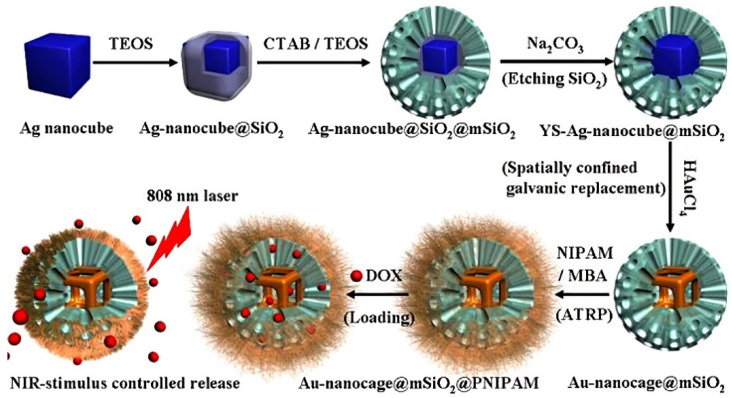
Schematic illustration of the NIR light-responsive Au-nanocage@mSiO_2_@PNIPAM for controlled drug release. Reprinted/adapted with permission from Ref. [81]. Copyright 2013, American Chemical Society.

Dual stimuli of acidic pH and NIR light have been considerably explored to date. Yang et al. [81] established an NIR-responsive controlled-release DDS (Au-nanocage@mSiO_2_@PNIPAM nanocomposites) based on AuNCs with mesoporous silica (mSiO_2_) shells as a carrier for enhanced drug loading, and poly(N-isopropylacrylamide) (PNIPAM) as an NIR-responsive gatekeeper. Upon NIR light irradiation, the AuNC core could efficiently convert photon energy into heat, which caused the thermally responsive PNIPAM covering the exterior of mSiO_2_ to collapse, exposing the pores of the mSiO_2_ shell and allowing the release of trapped DOX (Figure 8). Notably, the release of DOX increased with decreasing pH under NIR light irradiation, due to the fact that positively charged DOX molecules were loaded into the nanocomposites via electrostatic attraction with negatively charged mSiO_2_ channels. The electrostatic interaction weakened with decreasing pH, causing more DOX molecules to be released. Because tumor tissues have lower pH values than normal tissues, such light- and pH-responsive releases are applicable to cancer treatment.

**Figure 9:**
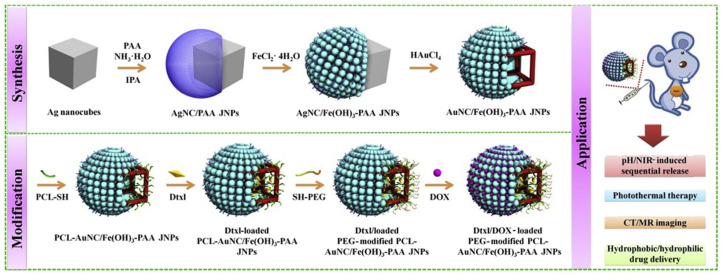
Schematic illustration of the fabrication, modification, and application of pH- and light-responsive AuNC/Fe(OH)_3_-PAA Janus nanoparticles. Reprinted/adapted with permission from Ref. [82]. Copyright 2018, Elsevier Ltd.

In another study, Zhang et al. [82] synthesized a multifunctional poly(3-caprolactone)-gold nanocage/ferric hydroxide-poly(acrylic acid) (PCL-AuNC/Fe(OH)_3_-PAA) dual drug delivery system (Figure 9). The hydrophobic AuNC side could be loaded with docetaxel (Dtxl), while the negatively charged Fe(OH)_3_-PAA side could be loaded with positively charged DOX. Because polyacrylic acid (PAA) is a pH-sensitive polymer and Fe(OH)_3_ nanoparticles are unstable in acidic media, DOX can be partially released from fragmented Fe(OH)_3_ in response to acidic pH excitation. Under weak NIR light irradiation (0.5 W/cm^2^), the internal heat generation of AuNCs was greater than that of Fe(OH)_3_-PAA, which leads to the increased solubility of Dtxl in AuNCs, the accelerated discharge of Dtxl, and the thiolated PCL can be isolated from the nanohybrid, but it had little effect on DOX release from the Fe(OH)_3_-PAA sector. Under the irradiation of a higher-intensity NIR laser, AuNCs and Fe(OH)_3_-PAA were stimulated to generate more heat, resulting in the simultaneous release of Dtxl and DOX. Selective sequential release of Dtxl and DOX was achieved using nanohybrids with independent pH and NIR sensitivity, and the synchronized release of both drugs improved the therapeutic effects by 5%. Furthermore, the outstanding computed X-ray tomography/magnetic resonance (CT/MR) imaging abilities of AuNCs and Fe(OH)_3_ showed that the Janus nanoparticles (JNPs) might efficiently guide cancer treatment. Additionally, under NIR light irradiation, mice administered with the dual drug-preloaded PCL-AuNC/Fe(OH)_3_-PAA JNPs displayed improved tumor inhibition compared to solo drug, cocktail, and dual drug treated mice, demonstrating the efficacy of combined cancer treatment.

Acid-degradable inorganic materials can act as gatekeepers for controlled drug release, providing an opportunity to design pH-responsive DDSs. Shi et al. [83] prepared a smart therapeutic nanoplatform based on Fe_3_O_4_@CaP-capped AuNCs. After the nanoplatform was internalized by cancer cells through endocytosis, CaP was degraded into the ionic state (Ca^2+^, PO_4_^3−^) in the acidic environment of endosomes and lysosomes, and the blocker Fe_3_O_4_@CaP was turned on to trigger drug release. In addition, upon NIR light irradiation, the heat generated by AuNCs not only ablated cancer cells, but also promoted the release of DOX, thus improving the therapeutic effect of chemotherapeutic drugs. Therefore, the light- and pH-responsive therapeutic nanoplatform achieved the synergistic effect of chemotherapy and photothermal therapy, showing better cell-killing efficacy.

### 4.2. Boolean Logic Gate-Regulated Double Light-Responsive AuNCs

**Figure 10:**
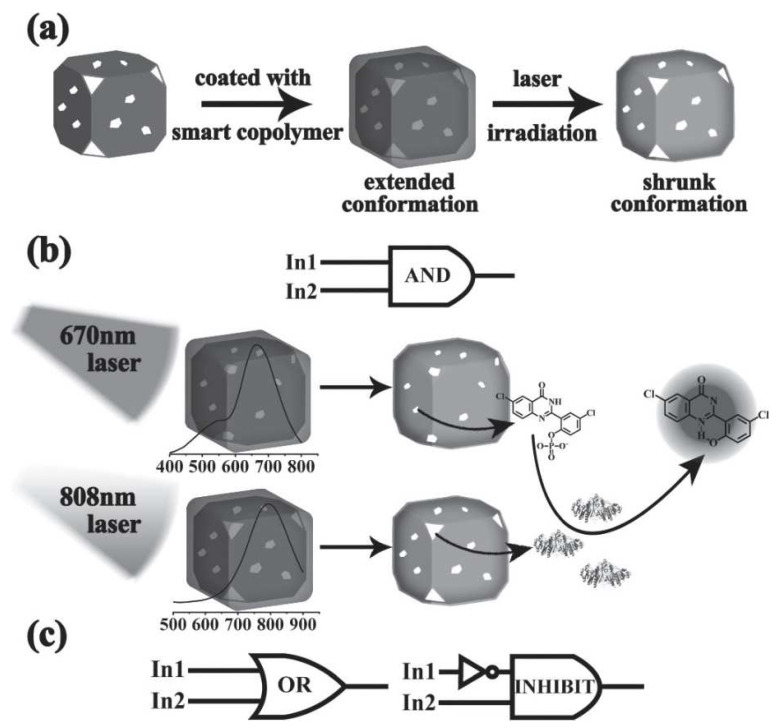
(**a**) Illustration of light-responsive AuNC copolymer. (**b**,**c**) Schematic diagram of two wavelengths of light (808 nm and 670 nm)-encoded logic gates for controlled release based on AuNC copolymer. Reprinted/adapted with permission from Ref. [91]. Copyright 2013, John Wiley & Sons, Inc.

Boolean logic systems based on molecular gates are receiving increasing attention [84,85]. The molecular gates of these smart devices, similar to digital electronic logic gates on silicon chips, can intelligently respond to various stimuli based on Boolean operations [86]. With their intelligent judgment ability, computing systems can perform complex operations, accurately process multiple data streams (inputs) from complex environments, and automatically generate responses (outputs). These breakthroughs have accelerated the development of more advanced controlled-release nanostructures [87,88]. Many research groups have applied the concept of logic gates to the design of intelligent delivery systems [89,90]. Among them, Shi et al. [91] prepared AuNCs with LSPR peaks at 808 nm and 670 nm, both enveloped by poly(N-isopropylacrylamide-co-acrylamide). The two AuNCs were loaded with an enzyme (alkaline phosphatase) and its substrate, respectively. When simultaneously illuminated by a matched laser beam as an input signal, both AuNCs generated heat to open the pores, releasing the enzyme and substrate that can interact and output an “AND” logic fluorescent signal. They also loaded isoenzyme or enzyme inhibitors into the AuNCs, to realize “OR” or “INHIBIT” logic gates, respectively (Figure 10). These designs will provide new insights into the development of other logic-controlled DDSs.

### 4.3. Light- and Glutathione-Responsive AuNCs

**Figure 11:**
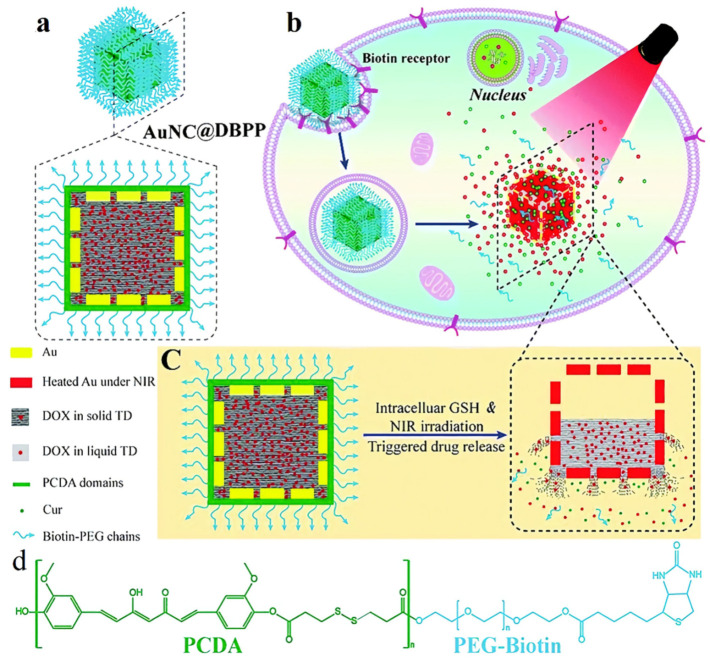
(**a**) Schematic diagram of AuNC@DBPP. (**b**,**c**) Light- and glutathione-responsive endocellular drug release, and (**d**) molecular structure of BPP. Reprinted/adapted with permission from Ref. [94]. Copyright 2017, The Royal Society of Chemistry.

Glutathione (GSH) is a tripeptide whose content in the cytoplasm (about 2–10 mM) is 2–3 orders of magnitude higher than that in the extracellular fluids (about 2–20 μM) [92]. Therefore, GSH is considered to be an ideal endogenous stimulant, which can quickly destabilize certain nanocarriers in cells, thus achieving effective intracellular drug release. This targeted intracellular drug release approach can significantly improve drug efficacy and reduce side effects related to drugs and nanocarriers [93]. In one study, Zhang et al. [94] developed a combination of light- and GSH-responsive strategies, wherein AuNC@DBPP nanoparticles were successfully fabricated by filling the cavity of AuNCs with DOX-containing 1-TD, followed by surface conjugation of polycurcumin (Figure 11). At body temperature and at low concentrations of GSH (5 μM) in the blood, the nanosystem released a small amount of drug. However, NIR light radiation at 5.0 W/cm^2^ and a high concentration of GSH at 5 mM (intracellular level) induced the release of DOX. Thus, this design successfully demonstrated dual-stimuli-responsive characteristics of the prepared nanodevices. Due to the NIR light and redox responsiveness of the nanosystem, as well as the combined effect of DOX and Biotin-PEG-Poly(curcumin-dithiodipropionic acid), (BPP, which acted as a chemosensitizer), AuNCs@DBPP showed good cytotoxicity to MCF-7/ADR cells and significantly caused cell death under NIR light irradiation. This controlled-release drug delivery system, triggered by both endogenous and exogenous stimuli, can minimize the side effects caused by premature drug release and then maximize the therapeutic effect.

### 4.4. mRNA- and Light-Responsive AuNCs

Cancer is often accompanied by changes in messenger RNA (mRNA). The loss of function of many tumor suppressors is caused by abnormal mRNA, so tumor-related mRNA can be used to trigger the release of drugs from carriers [95]. For example, C-myc mRNA, which exists in a variety of cancers, is particularly important in the development and progression of breast cancer [96]; TK1 mRNA is related to cell division and has been considered a tumor growth marker [97]; GalNAc-transferase mRNA (abbreviated as GalNAc-T mRNA, or GT mRNA) is abundantly expressed in many cancer cells and plays a vital role in the synthesis of gangliosides GM2/GD2 [98,99,100].

**Figure 12:**
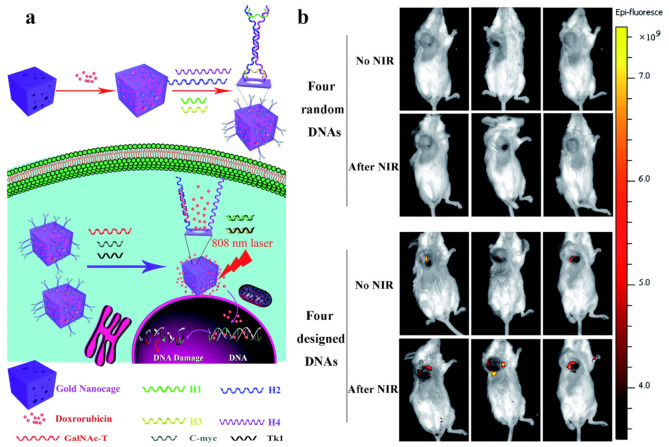
(**a**) Schematic illustration of the mRNA- and light-responsive AuNCs for controlled drug release; (**b**) in vivo fluorescence images of tumor-bearing mice injected with ICG-loaded AuNCs. Reprinted/adapted with permission from Ref. [101]. Copyright 2021, The Royal Society of Chemistry.

Zhang et al. [101] loaded DOX into the cavities of AuNCs and modified the triple-interlocked I-type DNA nanomodule on the surfaces of the AuNCs to achieve the closure of the AuNCs’ holes. By simultaneously combining three kinds of tumor-associated mRNAs (i.e., C-myc mRNA, TK1 mRNA, and GT mRNA) in breast cancer cells, the triple-interlocked nanomodule could be unlocked for precise drug release. However, in other cells that do not possess the three kinds of mRNA simultaneously, full-strand hybridization replacement could not be carried out and the drug could not be released. Additionally, a thermal effect was achieved when the NIR light was used to illuminate the AuNCs. The residual medicines could be pushed out of the AuNCs by the heat generated by the NIR light (Figure 12a). In addition to the dual-stimuli-regulated drug release, the DDS also successfully exhibited accurate imaging and efficient photothermal therapy, making it a very promising multifunctional nanoplatform for tumor diagnosis and treatment. Furthermore, they conducted a negative control experiment in vivo, in which AuNCs were locked by four kinds of random DNAs, to test the specificity of the triple-interlocked DDS. The NIR fluorescence probe ICG was employed to substitute the DOX filled in the AuNCs in order to analyze drug release in mice by in vivo fluorescence imaging. To evaluate the controlled-release ability in vivo, they administered AuNCs into mice tumors and then used a whole-body imaging system to detect the fluorescence of ICG four hours later. As shown in Figure 12b, the fluorescence intensity of the ICG locked by the four kinds of random DNAs was weaker than that of the ICG locked by the I-type DNAs employed in this experiment. Since the four kinds of random DNAs could not be unlocked by the target mRNAs, ICG was rarely released from the random-DNA-locked AuNCs, indicating that the triple-interlock device was effective in vivo. In addition, four hours after injection, they detected faint ICG fluorescence in mice given AuNCs locked with I-type DNAs. Upon NIR irradiation, the fluorescence intensity of ICG was enhanced. This also proved that the drug release in vivo is not only controlled by mRNAs, but also driven by NIR laser-induced photothermal effects.

### 4.5. ATP- and Enzyme-Responsive AuNCs

Because both stimuli naturally exist in certain specific tumor sites, ATP and enzyme dual-responsive AuNCs have been proposed and synthesized.

Employing AuNCs coated with DNA molecular gates, a new controlled-release biosensor for ATP isothermal amplification detection was developed and tested in intracellular ATP detecting. Two types of thiolated DNAs, S1 and S2, were modified on the AuNCs surface via Au–S bonds. Each molecular gate was generated by hybridizing a long-stranded DNA S3 with two fixed SH-DNAs. The molecular gates could prevent the discharge of fluorescent molecules such as RhB filled in the cavities of AuNCs. The primer S4 was used to serve as a recognition moiety. Due to the specific binding of ATP and ATP aptamers, the primer S4 was liberated from the double-stranded hybridization with ATP aptamers. With the intervention of DNA polymerase and nicking endonuclease, the liberated S4 will launch the autonomous replication–scission–displacement pathway. To achieve cyclic enzymatic amplification of the discharge of guest molecules from AuNCs, the DNA S3 was designed to involve an Nb.Bpu10I nicking endonuclease recognition sequence as well as a sequence complementary to the primer S4. The constructed nanodevice was proved to be a reliable biosensor for both qualitative and quantitative detecting of target molecules [102].

**Figure 13:**
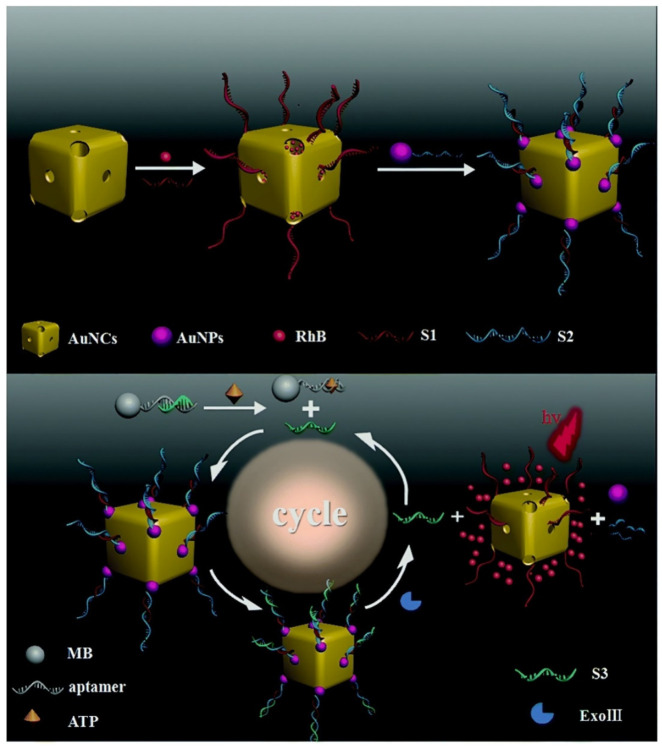
Schematic illustration of the AuNC-based ATP- and enzyme-responsive biosensor system for the detection of ATP. Reprinted/adapted with permission from Ref. [103]. Copyright 2020, The Royal Society of Chemistry.

Wang et al. [103] designed an AuNC-based ATP and Exonuclease III (Exo III)-responsive fluorescent biosensor. Gold nanoparticles were used as building blocks in the system to cover the pores of RhB-loaded AuNCs via DNA hybridization. Under the stimulation of ATP and Exo III, RhB molecules were finally released for detection (Figure 13). The biosensor had a linear ATP detection range of 1.0 × 10^−6^ to 1.0 × 10^−4^ mM, with a detection limit of 0.88 nM. It could also distinguish between ATP and ATP analogs such as guanosine triphosphate, cytidine triphosphate, and uridine triphosphate because of its high selectivity for ATP. The proposed strategy could be further extended to treatment systems with multiple functions such as molecular imaging, chemotherapy, and photothermal therapy.

### 4.6. pH-, Light-, and Enzyme-Responsive AuNCs

**Figure 14:**
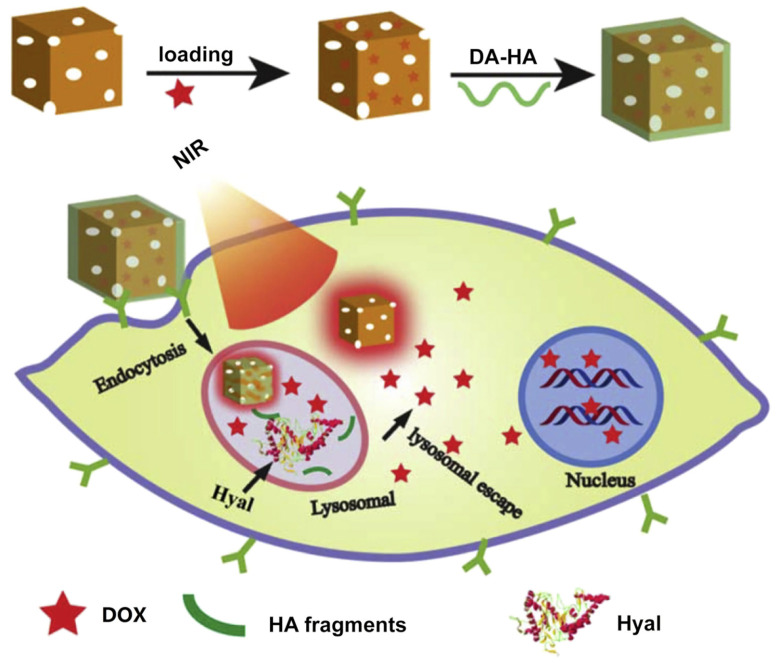
Schematic illustration of a multi-stimuli-responsive nanoplatform based on AuNCs-HA loaded with DOX to precisely control intracellular drug release and synergistic therapy. Reprinted/adapted with permission from Ref. [105]. Copyright 2014, Elsevier Ltd.

In addition to dual-responsive AuNCs, several multi-responsive AuNCs have been developed recently. Zhan et al. [104] fabricated a triple-stimuli sensitive hybrid nanodrug (EA-AB, in which EA and AB represent erlotinib-loaded AuNCs and AuCluster@BSA, respectively. BSA = bovine serum albumin) for controlled release of drugs, photothermal therapy, and fluorescent and multispectral optoacoustic tomography imaging. For this nanodrug (EA-AB), EA were capped and functionalized by AB via electrostatic interaction. After cellular internalization, low-pH and lysosomal proteases induced the release of erlotinib from EA-AB, allowing the AuCluster to restore its fluorescence for imaging. NIR light irradiation further promoted drug release and exerted the effect of photothermal therapy. To precisely release drugs in target cells, Wang et al. [105] prepared multi-stimuli-responsive nanohybrids (AuNCs-HA) based on AuNCs and hyaluronic acid (HA). Through CD44 receptor-mediated interactions, the nanohybrids could be effectively endocytosed. Subsequently, the HA on the surfaces of nanohybrids could release the loaded DOX only after being degraded by Hyal in cells (Figure 14). Simultaneously, acidic pH and NIR stimulation might efficiently enhance DOX release, significantly enhancing the therapeutic effect and reducing drug toxicity. Furthermore, the combination of chemotherapy and photothermal therapy completely inhibited tumor development in vivo as compared to the two therapies separately. Based on HA-modified AuNCs, our group further decorated PEG [106], thermoresponsive copolymer P (NIPAM-co-Am) [107], and liver cancer-specific adhesion peptide [108] on AuNCs, thus obtaining a variety of DDSs that simultaneously respond to light, pH, and enzymes, allowing for the combined treatment of tumors. In summary, these findings may promote the development of non-invasive and precise drug delivery systems to reduce the nonspecific systemic diffusion of toxic drugs and maximize tumor-targeted drug-delivery efficacy.

## 5. Conclusions and Perspectives

With the rapid development and interpenetration of nanotechnology and biomedicine, a variety of AuNC-based stimuli-responsive DDSs are being explored for the diagnosis and treatment of cancers. In this review, we summarized the progress of AuNC-based nanomaterials as stimuli-responsive controlled-release systems. AuNCs can be applied in stimuli-responsive DDSs, mainly based on their adjustable particle size, large specific surface area, excellent drug-loading capacity, good biocompatibility, and easy functionalization. Stimuli-responsive AuNCs are able to remain stable in the circulatory system and, upon reaching the lesion site, release their payload in response to endogenous or exogenous stimuli, thereby improving the therapeutic efficacy and reducing side effects. Among them, dual/multi-stimuli-responsive AuNCs (e.g., pH and light dual-stimuli; light and GSH dual-stimuli; pH, light, and enzyme multi-stimuli) can better respond to the environment of cancer cells and achieve higher specificity and efficacy. Delivering combinations of multiple therapeutic agents also makes sense because systems this can effectively provide multiple treatments, can overcome multidrug resistance, and synergistically enhance therapeutic efficacy. By combining imaging agents with nanoplatforms, key characteristics of tumors can be visualized, thus helping to personalize cancer therapy. Nanosystems with imaging capabilities can enhance the traceability of drug delivery systems in vivo, thereby facilitating understanding of their interactions with organisms and helping to optimize the design of DDSs.

Nevertheless, most studies of stimuli-responsive AuNCs are still in the proof-of-concept stage, far from clinical application. To translate the proof-of-concept studies into approved DDSs, not only the specific problems for each type of stimulus mentioned above need to be addressed, but also other common problems of DDSs need to be overcome.

Firstly, most proof-of-concept studies usually can not completely solve the problems related to the feasibility of their clinical application. For instance, interindividual variation, heterogeneity of tumors, and changes in metabolic levels may lead to fluctuations of endogenous stimuli signals and the failure to release cargo on demand. As for the external stimuli-responsive DDSs, issues such as whether the required penetration depth can be achieved, whether the impact on surrounding normal tissues can be reduced, and whether the exposure duration is safe need to be focused on. Furthermore, to our knowledge, no AuNC-based DDSs have entered the clinical research stages. Even if DDSs show excellent performances in animal trials, it is not sufficient to show that the systems will show superior results to existing clinical therapies, as preclinical trials are typically carried out in solid tumor models, but metastatic tumors are the main cause of cancer-related deaths. DDSs with good effect in solid tumor models might be ineffective in the treatment of metastases. In addition, tumor models established in rodents grow rapidly and have good EPR effects, while tumors that grow naturally in patients have more significant heterogeneity and weaker EPR effects.

Secondly, the design and preparation of stimuli-responsive nanocarriers are complicated, which will lead to difficulties in large-scale manufacturing and limit their potential for clinical translation. Compared with single-stimulus-responsive AuNCs, dual- and multi-stimuli-responsive AuNCs have more fantastic performances. However, their more complex design and preparation processes make them more challenging to enter the clinical research phases, so their multifunctionality and complexity should be carefully weighed.

Thirdly, compared with ordinary AuNCs, the safety and efficacy of stimuli-responsive ones are more likely to vary between individuals. Among the finite number of stimuli-responsive AuNCs, the studies on their toxicity are very limited, so more systematic toxicological studies (such as long-term toxicity, neurotoxicity, and genotoxicity) need to be carried out.

In addition, economic efficiency, immunogenicity, and ways of elimination from the body are issues that need attention in developing various DDSs, including AuNCs.

In summary, all of these issues pose challenges to the clinical translation of stimuli-responsive AuNCs. These problems require continuous efforts, as well as close interdisciplinary cooperation and industry-university-research cooperation, to ensure the clinical transformation of these intelligent stimuli-responsive nanodevices in the future.
